# Microbial communities from arid environments on a global scale. A systematic review

**DOI:** 10.1186/s40659-020-00296-1

**Published:** 2020-07-06

**Authors:** Javiera Vásquez-Dean, Felipe Maza, Isidora Morel, Rodrigo Pulgar, Mauricio González

**Affiliations:** 1 Laboratorio de Bioinformática y Expresión Génica, Instituto de Nutrición y Tecnología de los Alimentos, Universidad de Chile, Santiago, Chile; 2 Center for Genome Regulation (CGR), Santiago, Chile; 3 Laboratorio de Genómica y Genética de Interacciones Biológicas (LG2IB), Instituto de Nutrición y Tecnología de los Alimentos, Universidad de Chile, Santiago, Chile; 4 Scimetrica Lab, Santiago, Chile

**Keywords:** Arid soil, Microbiomes, NGS, Systematic review, Global-scale

## Abstract

Arid environments are defined by the lack of water availability, which is directly related to the mean annual precipitation (MAP), and high values of solar irradiation, which impacts the community composition of animals, plants, and the microbial structure of the soil. Recent advances in NGS technologies have expanded our ability to characterize microbiomes, allowing environmental microbiologists to explore the complete microbial structure. Intending to identify and describe the state-of-the-art of bacterial communities in arid soils at a global scale, and to address the effect that some environmental features may have on them, we performed a systematic review based on the PRISMA guideline. Using a combination of keywords, we identified a collection of 66 studies, including 327 sampled sites, reporting the arid soil bacterial community composition by 16S rDNA gene high-throughput sequencing. To identify factors that can modulate bacterial communities, we extracted the geographical, environmental, and physicochemical data. The results indicate that even though each sampled site was catalogued as arid, they show wide variability in altitude, mean annual temperature (MAT), soil pH and electric conductivity, within and between arid environments. We show that arid soils display a higher abundance of Actinobacteria and lower abundance of Proteobacteria, Cyanobacteria, and Planctomycetes, compared with non-arid soil microbiomes, revealing that microbial structure seems to be strongly modulated by MAP and MAT and not by pH in arid soils. We observed that environmental and physicochemical features were scarcely described among studies, hence, we propose a reporting guideline for further analysis, which will allow deepening the knowledge of the relationship between the microbiome and abiotic factors in arid soil. Finally, to understand the academic collaborations landscape, we developed an analysis of the author’s network, corroborating a low degree of connectivity and collaborations in this research topic. Considering that it is crucial to understand how microbial processes develop and change in arid soils, our analysis emphasizes the need to increase collaborations between research groups worldwide.

## Background

About 40% of the world’s total land surface is classified as dryland soils according to the United Nations Committee to Combat Desertification (UNCCD). Within dryland environments, arid and hyper-arid are the driest, characterized by a very low ratio of Mean Annual Precipitation (MAP) to Potential Evapotranspiration (PET; MAP/PET) [28], a high level of solar radiation and a restricted vegetation cover [10]. In the soil microbiome, bacteria are the most abundant and diverse organisms [17], and their presence is crucial for plant growth [2]. As arid environments display scarce vegetation, the presence and abundance of bacteria are essential for nutrient cycling and carbon storage [4, 11].

A meta-analysis of soil microbial communities shows that biogeography of microorganisms is driven by variables that are different to those of macroorganisms [12], and microbial communities are structurally dependant on local factors rather than large scale factors like geographical isolation [35]; Skujinš [37, 42]. Although much is known about matters related to soil bacterial communities, we do not know the magnitude of the body of knowledge, nor do we fully understand the ecology of these communities in dryland ecosystems [25, 34]. Unveiling the patterns of bacterial communities from arid soils is essential for improving Earth system models [46], which are relevant for projecting the effects of phenomena as desertification [41]; Yhao Yongping et al. [47].

Next-generation sequencing (NGS) description of soil communities has grown broadly, and initiatives like the Earth Microbiome Project [42] have arisen to increase our global-scale understanding of environmental microbiomes. Many independent researchers have sought to describe the arid soil bacterial communities studying individual arid environments [19, 26] and some have compared a couple of them [16, 27], but none have managed to compare arid environments globally.

Data is accumulating rapidly, but a systematic collection of articles studying bacterial communities reported from arid soils has not been performed. This published data review would give us insight into the global landscape of arid soil microbiology and permit us to identify gaps in the body of knowledge. That is precisely the focus of this review. Based on the PRISMA guidelines [24] we aimed to perform a multi-approach systematic review to identify all, or most, of the publications that address the goal of describing the arid soil microbiota by NGS strategies. Then, from each retrieved study, we extracted data and performed qualitative analyses over the relative abundance of bacteria at the phylum level, environmental features, and the academic information associated with the studies, all of which are further described and discussed.

## Methods

### Information sources and search

This systematic review followed the PRISMA guidelines [24]. We performed a keyword search in three electronic databases: PubMed, SCOPUS, and Web of Science (WOS). In the first step, databases were searched using a defined set of keywords, restricting the search engine to the title and abstract of the articles. The search was run on 15/05/2019, using the following search line: (1) soil* AND bacteri*AND communit* AND arid* AND [McMurdo OR Antarctica OR Arctic]. The results were compiled and submitted to the revision of titles and abstracts, which allowed preselecting articles that fit our inclusion criteria. In a second step, we reviewed the full text of these publications and selected those studies that certainly fit our inclusion criteria. To gather any articles missed by the keyword search, we included a third and final step, that consisted of scanning the reference list and corresponding authors’ publications of all the included studies.

### Eligibility criteria

Each article included in this review had to meet the following criteria: (1) must be an original research study, published in a scientific journal, available in English; (2) must have the information that describes bulk arid soil bacterial communities (0–20 cm belowground), from arid or hyper-arid environments, based in the definition described by Trabucco and Zomer [43]; (3) must have bacterial community data, described through the extraction of total genomic DNA directly from bulk soil samples and amplification and sequencing of the 16S rDNA gene using NGS technologies and (4) must contain soils extracted from unmanipulated/control/no human-impacted environments.

### Study selection

**Fig. 1:**
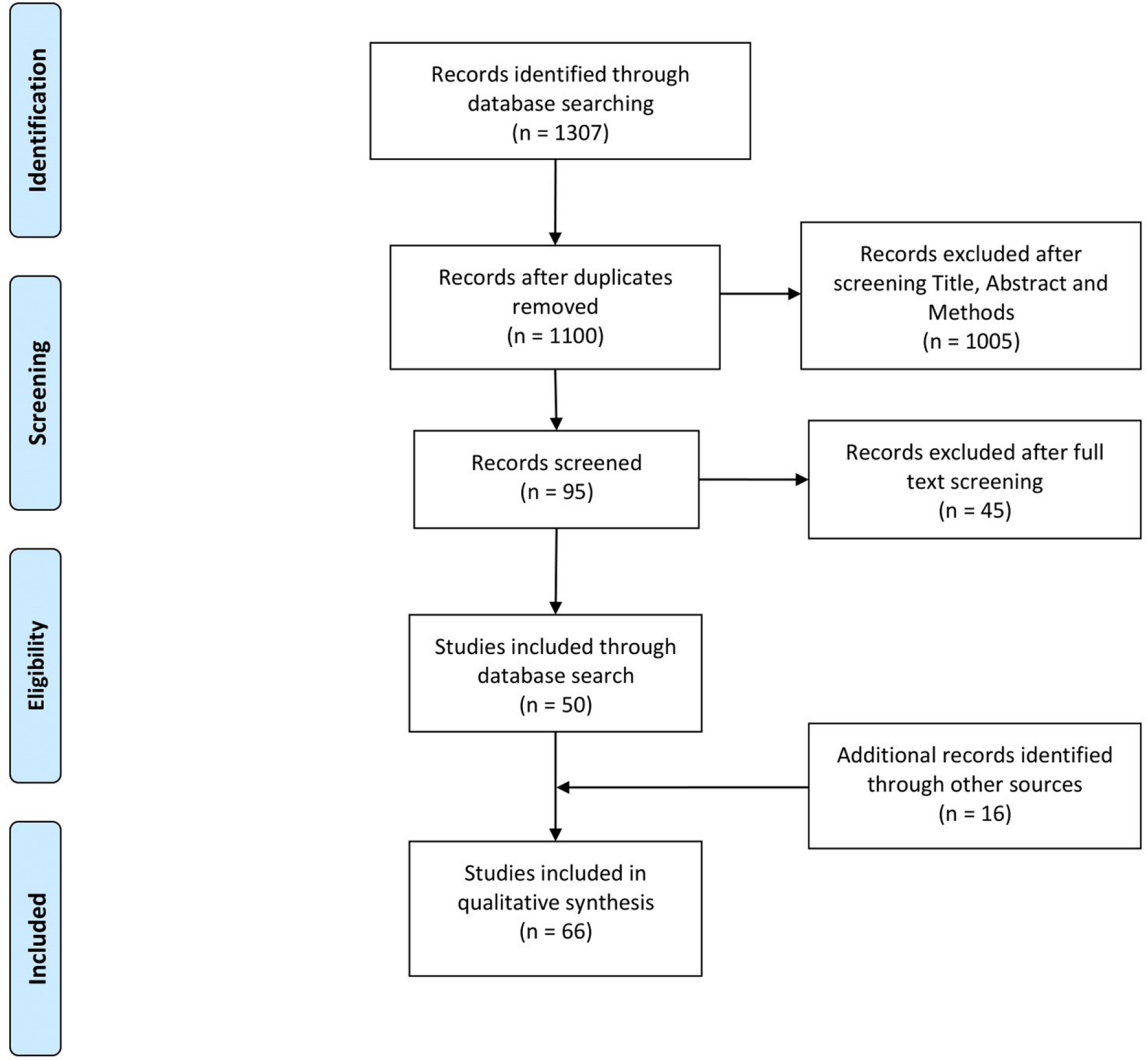
PRISMA Flow Diagram of information through the different phases of the systematic review

To avoid bias and loss of information, the eligibility assessment was performed systematically by three reviewers/authors (I.M., F.M., and J. V-D.). The articles selection was guided by the eligibility criteria previously stated. Disagreements between reviewers were resolved by reviewing the full article. When the three authors did not reach consensus over a specific article, an arbitrating reviewer (R.P.) was introduced. The PRISMA [24] flow diagram (Fig. 1) shows the number of articles in each step of the article selection process. To evaluate the search strategy, we calculated metrics such as Precision, Sensitivity, and Number Needed to read (NNR), before finding a relevant article in the output [13].

## Data extraction

The three aforementioned reviewers extracted the editorial, geographical, environmental, physicochemical, and methodological data of each selected study. This information was reviewed, agreed, and deposited in extraction sheets (Additional file 1: Table S1). The first sheet was used for editorial data from each article, including the following items: the journal where the article was published and the year of publication. The second sheet was used to extract geographical, environmental, physicochemical, and methodological data from each sampling site, including Global Positioning System (GPS) information, altitude (m a.s.l.), soil type, sampled depth (cm), soil pH, Mean Annual Temperature (MAT;  °C/year), Mean Annual Precipitation (MAP; mm/year), soil evapotranspiration (mm/year), soil conductivity (EC; µS/cm), Total Organic Carbon percentage (TOC), Total Nitrogen percentage (TN), soil texture, biome sampled [31], hemisphere sampled, aridity index of the environment [43], the protocol for DNA extraction, 16S rDNA gene variable sequence amplified, sequencing platform, software for sequence analysis and taxonomic affiliation database used. The third sheet was used to extract bacterial community relative abundance data of sampled sites at the phylum level and alpha diversity index (Shannon index; H’) of each sample. The biome and Aridity index were extracted from databases using the GPS information from each sample. When needed, reviewers searched articles referenced as methodology, to extract useful data of sampled sites. In order to compare global-scale arid soil bacterial communities to non-arid soil communities, we extracted community composition data from the global-scale meta-analysis performed by Bahram and colleagues [3].

### Editorial data analysis and co-authorship network assembly

To assess the academic landscape we plotted editorial data as publishing year, and journal impact factor from 2018 using the ggplot2 package in R and assessed the academic nexus through a co-authorship network using Social Network [22] in Cytoscape software [36]. The network was created by entering the title of the 66 articles included in this review in the PubMed query bar within the Social Network App. The network was then automatically created. The yFiles Organic layout was applied to improve network visualization. We used the NetworkAnalyzer tool in Cytoscape software to observe simple parameters of author networks as Connected components or number of module networks, the number of nodes/authors, Neighbour connectivity distribution, and power-law fit, Clustering coefficient, Network density and Betweenness Centrality of authors.

### Environmental and bacterial community data analysis

Extracted data were qualitatively described by plotting the environmental features of arid soils and bacterial phyla relative abundance of soil communities sampled by each report included. To visualize associations between environment features and microbial community structure, we plotted the community composition in stacked bar plots ordered according to different environmental features in R software using the ggplot2 package (Kahle and Wickham [21]).

## Results

### Search strategy analysis

To identify all (or most) global studies that address the description of “arid soil microbiota by NGS”, we performed a systematic review following the PRISMA guidelines. The keyword search from PubMed, Scopus, and WOS databases retrieved a total of 1307 records (Fig. 1). After removing redundancy, 1100 articles remained, which were screened through their title and abstract. Only 95 articles passed the established inclusion criteria and were then screened through the entire text. Forty-five of these articles did not meet the inclusion criteria, leaving us with a body of 50 articles. Also, 16 additional studies met the inclusion criteria after scanning the references and corresponding author’s publications of all the previously selected studies. This way, we defined a collection of 66 studies, including 327 sampled sites. Subsequently, to characterize the search strategy, we calculated metrics such as sensitivity, precision, and Number of Needed to Read (NNR). The sensitivity, defined as the proportion of relevant articles identified by the search strategy was 75%, while the precision, that describes the proportion of relevant studies identified as a percentage of all the records detected by the search strategy, was 4.5%. These results indicate a high sensibility and low precision, which is expected since systematic reviews with high sensitivity strategies incline towards low precision, which is reflected in the high NNR (22.2).

### Geographical, environmental and physicochemical features in arid soils and analysis of the information available in the selected studies

**Fig. 2:**
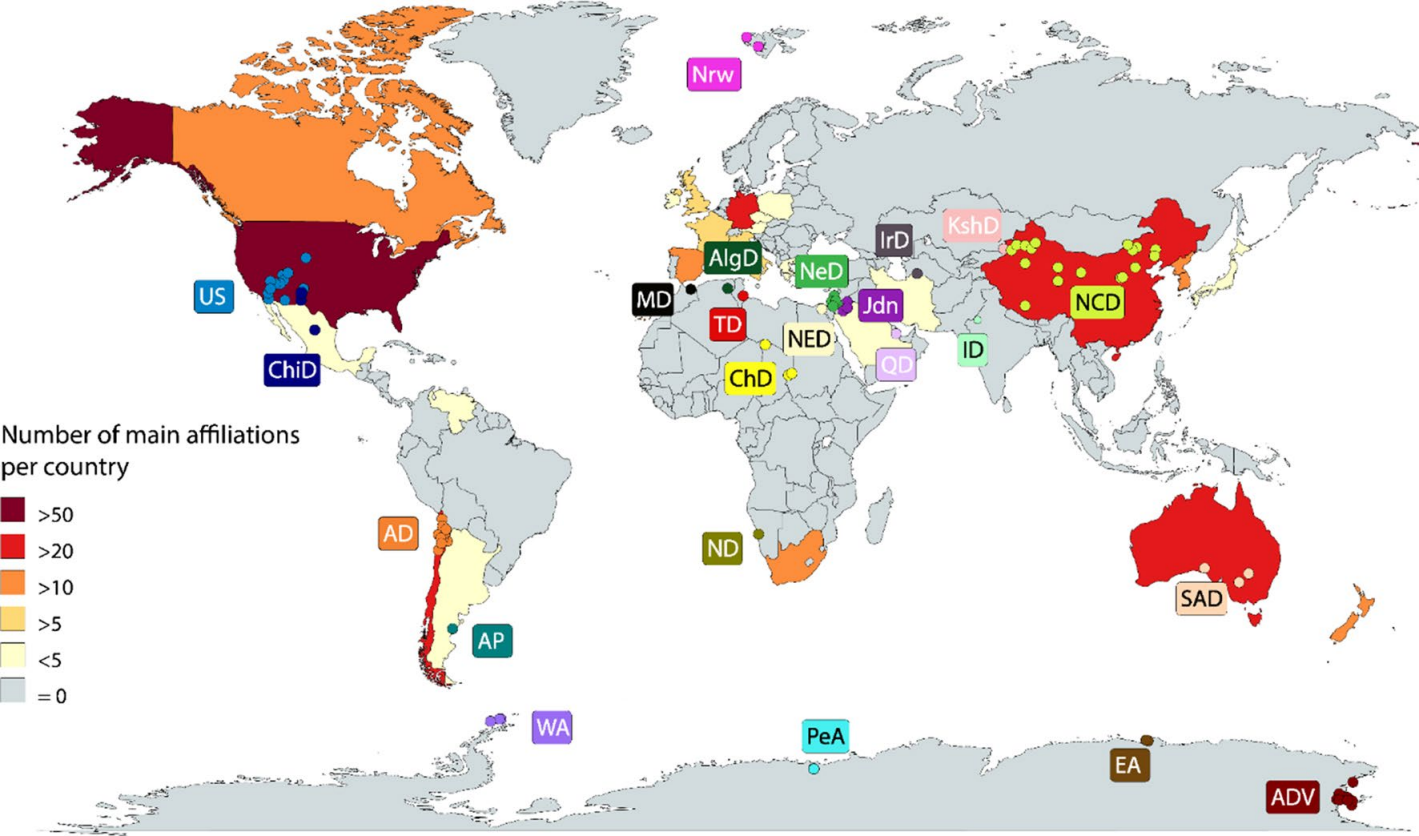
Worldwide arid environments studied. Dots depict 325 sampled sites from the 66 reports included in this systematic review, plotted using their GPS coordinates and ggmap tool from ggplot2 package in the R software [21]. Country colour denotes the number of main affiliations from each author in the articles included. Each arid environment sampled is depicted by a different colour, this map was built using the online tool Mapchart. Abbreviations: WA, Western Antarctica; AP, Argentinian Patagonia; AD, Atacama Desert; ChiD, Chihuahuan Desert; US, South Western United States; ND, Namibia Desert; PeA, Princess Elisabeth Station Antarctica; ChD, Chad Desert; MD, Moroccan Desert; AlgD, Algerian Desert; TD, Tunisian Desert; NED, North Egyptian Desert; NeD, Negev Desert; Jdn, Jordan arid soils; QD, Qatari Desert; IrD, Iran Desert; ID, Indian Desert; KshD, Kazakhstan Desert; NCD, North China Deserts; SAD, South Australia Desert; ADV, Antarctic Dry Valleys; Nrw, Norway

The complete set of data from each sample is showed in Additional file 1: Table S1 and summarized in Additional file 2: Table S2 by articles, for future researchers. The analysis of the available information shows that Geographical features of arid soils were reported in more than 50% of the studies. In terms of the sampling sites, 65 articles (98%) reported their GPS location coordinates, while only 41 (62%) reported their altitude (Additional file 3: Figure S1). To get a global perspective of the geographical distribution of studied sites, we plotted the GPS coordinates of each sample (Fig. 2). This graphical visualization highlights arid lands in every continent and every arid environment described by Trabucco and Zomer [43] has been sampled. However, the data density of sampled sites was not evenly distributed across arid environments. Out of all the sampled sites, the Antarctic Dry Valley (26%), North China Deserts (18%) and the Chilean Atacama Desert (16%) are the most sampled arid environments, while the Middle Eastern and African Deserts (6%) and the Argentinian Patagonia (0.3%) are the least sampled. We can also observe that arid soils are present in a wide range of altitudes, from 0 to 5000 m a.s.l (Additional file 3: Figure S2a). While some deserts contain the entire elevation range, 0 to 4900 m a.s.l. (Chilean Atacama and North China Deserts), others have low altitude variation (Antarctic Dry Valley and the Southwestern US Desert).

Regarding the environmental and physicochemical features, we observed that a considerable number of articles do not report these features for the studied sites. Within the environmental data, we looked for soil type, soil texture, sampling depth, Mean Annual Temperature (MAT) and Mean Annual Precipitation (MAP), while biome type and aridity index data were extracted from external databases [31, 43]. For physicochemical factors, we searched soil pH, soil conductivity, soil evapotranspiration, Total Organic Carbon percentage (TOC), and Total Nitrogen percentage (TN). Among the extracted features described, only the soil type, MAT, MAP, Soil conductivity, and TOC were reported in more than 50% of the studies. Other relevant environmental/physicochemical features such as sampling depth, soil texture, pH, TN, and soil evapotranspiration were scarcely reported (< 50% of the articles). The report of these data is relevant to determine the impact of these factors on the microbial composition of soils in arid zones.

Another type of environmental data extracted was biomes. Forty-four articles sampled environments classified as Desert and Xeric Shrubland, twenty-four as Tundra, two as Montane Grassland and Shrubland, and two as Temperate Grassland, Savannas, and Shrubland. Thus, these are the biomes with higher desertification risk. The MAP from these articles ranged from 0 to 400 mm/yr, the driest coming from the Chilean Atacama, Antarctic, and Middle eastern Deserts, while the less arid where the Southwestern US and North China Deserts. This last one also displayed the widest gradient of MAP within its sampled sites (Additional file 3: Figure S2b). Additionally, MAT also differed considerably across arid soils in general (Additional file 3: Figure S2c), ranging from − 22 °C to 30 °C, while more than 60% of the studied sites are in cold arid environments (CAE). The Chilean Atacama Desert displayed the widest range of MAT, which could be related to the altitude gradient and latitudinal extension of this environment. For these reasons the Atacama Desert, besides being the driest and having one of the highest altitude ranges and MAT, is an excellent place to study the effects of these factors on the structure of soil microbial communities.

Regarding the physicochemical aspects, arid sites exhibit an ample range of soil pH, being, in most cases, between 5 and 10 (Additional file 3: Figure S2d). The Antarctic Dry Valley (ADV) encompasses the widest range of pH in its soils, having two sites with pH less than 4. Moreover, ADV is also the arid environment with the widest range of soil conductivity. While most of the sites sampled in arid soils show conductivities less than 200 µS/cm, numerous soils sampled in ADV show conductivities up to approximately 1500 µS/cm (Additional file 3: Figure S2e), suggesting that ADV is a good place to study the effect of pH and soil conductivity on microbial communities. As expected, most of the arid soils sampled exhibited very low TN and TOC values (Additional file 3: Figure S2f, g), which is explained by the limited organic biomass in these environments.

### Methodological data

As the retrieved studies have been carried out by different research groups using different methods, the methodological variability used to characterize the microbial communities must be considered. Our results show that at least ten different DNA extraction protocols were used, being the PowerSoil DNA isolation kit the most used (36% of articles in at least one sample) followed by the FastDNA Spin Kit for Soil (24%, in at least one sample) and also in-house extraction methods (22.7%, in at least one sample). When addressing sequencing techniques, Roche 454 pyrosequencing was chosen 54% of the time, while the other 45% used the Illumina technology. The preferred variable region of the 16S rDNA gene sequenced to determine soil bacterial community composition was the V4 region (19%), followed by V3 (16%) and V5 (16%). The analysis of the sequenced reads was done by eleven different software/algorithms, Qiime (60.6%) and Mothur (27.2%) being the most used. Taxonomic affiliation of Operational Taxonomic Units (OTUs) was performed using the Ribosomal Database Project (RDP; 42.4%), Greengenes database (31.8%), and SILVA database (19.7%). Despite the variability of methodological strategies used to characterize the microbial communities of arid soils, we did not find an association between the methodological strategy and the variation in the structure of the analysed microbial communities.

### Arid soil bacterial community structure and diversity patterns

From the articles included in this systematic review, we also extracted the relative phyla abundance and the Shannon diversity index (H’). Similar to geographic and environmental data, a percentage of the articles (11%) did not report the exact values of phyla relative abundance and diversity index for each site sampled. Thus, we were able to extract the relative bacterial abundance of 32% of the samples and the diversity index from 46.1% of them.

Shannon diversity index (bacterial community alpha diversity) values ranged from 1.6 to 10.36 in the arid soil (Additional file 3: Figure S2h). Samples from The Sonoran Desert (US) displayed the highest H’ value, while samples from North China Deserts (NCD) showed the lowest H’ value. Alike, the results showed that there is high variability in the bacterial diversity within the arid environments, highlighting the Atacama and North China Deserts, which displayed samples that were ranging under two and over ten alpha diversity indices. Notably, most of the samples from the Antarctic Dry Valley (ADV) have a restricted diversity index range (between 4 and 6), which contrasts with the wide range of pH and conductivity. Thus, there does not seem to be an association between pH, conductivity, and microbial diversity in cold arid soils.

**Fig. 3:**
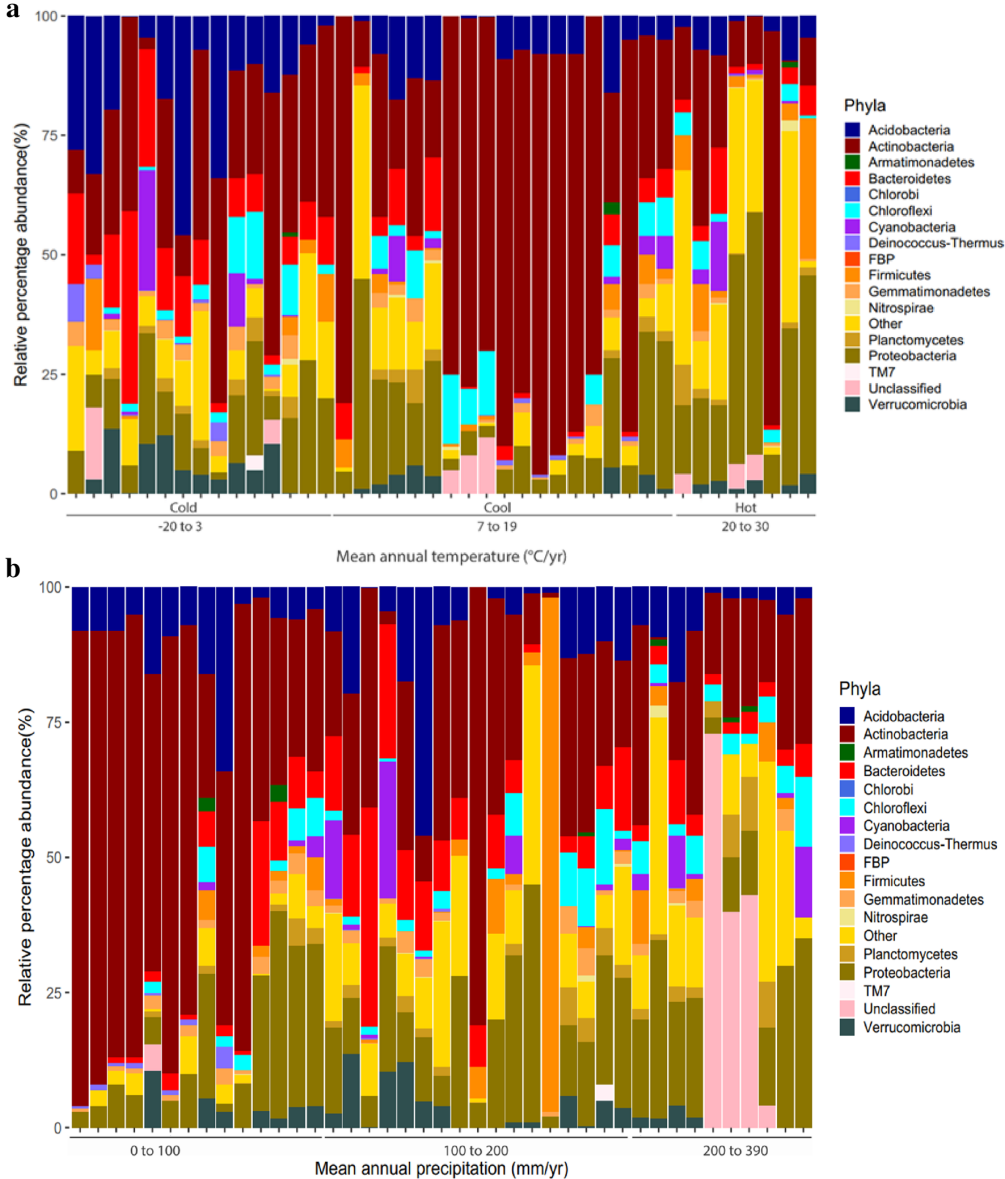
Community structure of arid soil bacteria varies with MAT and MAP. Vertical bars represent mean relative abundance (%) of each phylum in the samples from articles included in this systematic review. Bars indicate relative abundance coloured by bacterial phyla; **a**: Relative abundance (%) sorted according to mean annual temperature (MAT). **b** Relative abundance (%) sorted according to mean annual precipitation (MAP)

**Fig. 4:**
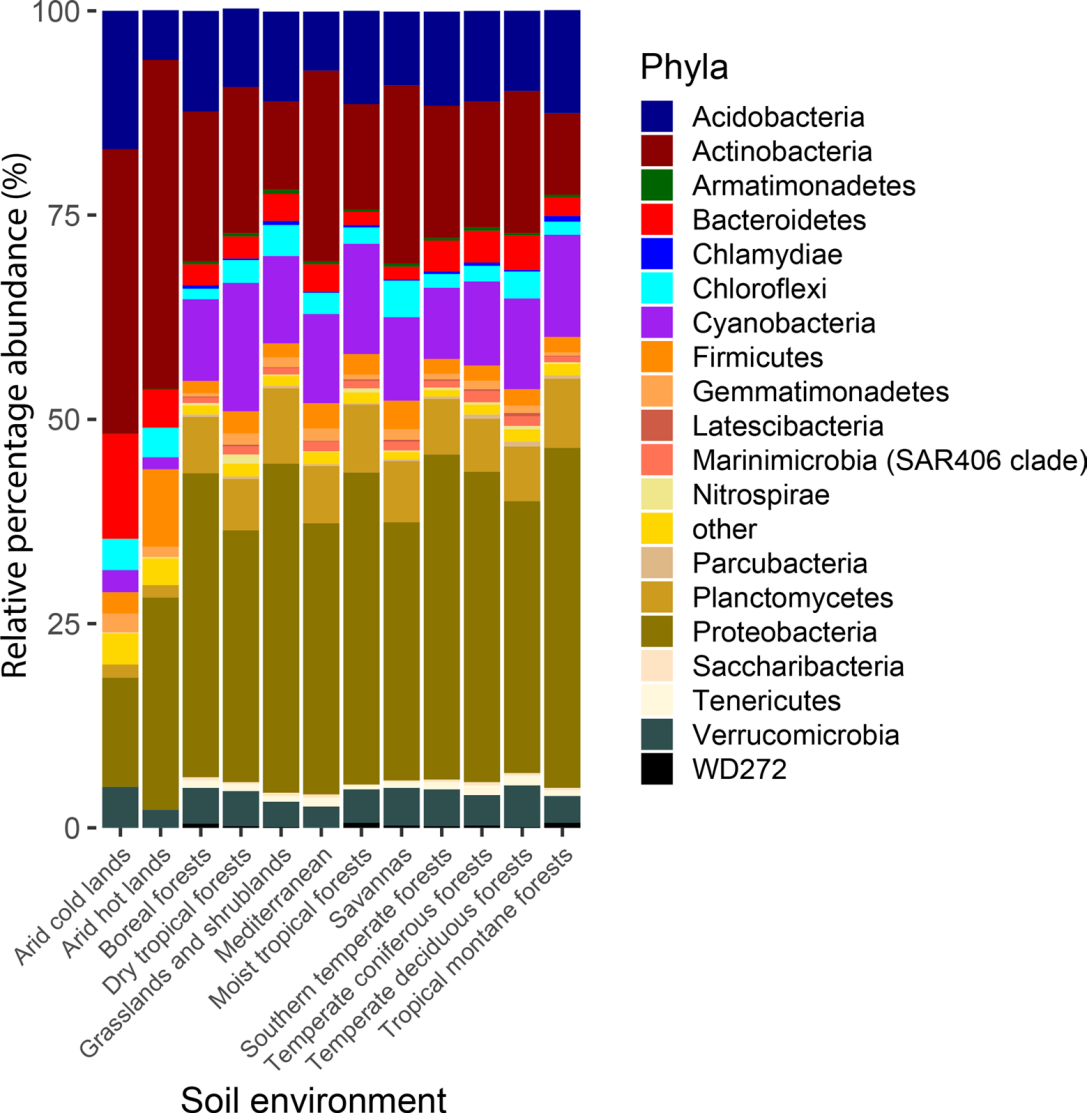
Bacterial communities of arid soil are taxonomically different from other soils. Two first vertical bars represent mean relative percentage abundance of samples from articles included in this systematic review as cold arid soils and hot arid soils, the rest of soil environments correspond to data reported in Bahram et al. [3]. Bars indicate relative abundance coloured by bacterial phyla. Plots where generated using ggplot2 package in R software

To analyse the bacterial community structure and its relationship with the environmental and physicochemical features described, we plotted the relative abundance of OTUs against some of these features. When plotting the relative microbial abundance against soil pH, we did not observe a pattern of the community structure associated with changes in this variable, confirming the observation that pH does not seem to be a strong driver for bacterial communities in arid soil (Additional file 3: Figure S3). In contrast, when we compare the average relative microbial abundance against MAT (Figs. 3a, 4), we observe an evident effect of this factor over some phyla. For instance, while soil communities of cold/cool environments are dominated by Actinobacteria (31.8%), Acidobacteria (15.4%), Proteobacteria (12.1%) and Bacteroidetes (11.7%), the soil communities of hot environments are dominated by Actinobacteria (36.8%), Proteobacteria (23.8%), Firmicutes (8.6%) and Acidobacteria (5.5%), showing a shift in the taxa distribution among the environments. Likewise, although Actinobacteria was the dominant phylum in arid soils as a whole, its relative abundance notably decreases when MAP increases (Figs. 3b, 4). These observations emphasize the impact that both temperature and water availability have over the relative abundance of some microbial taxa in arid soils.

To determine the similarities and differences between the structure of the arid and non-arid soil bacterial communities, we compared the relative abundance of phyla between the communities analysed in the present study with forest and others non-arid soil bacterial communities reported by Bahram and colleagues [3]; Fig. 4. Interestingly, in non-arid soils, bacteria from Marinimicrobia, Chlamydiae, Tenericutes and Saccharibacteria phyla are present, unlike in arid soils. Furthermore, we observed that arid soils had a higher abundance of Actinobacteria and a lower abundance of Proteobacteria, Cyanobacteria, and Planctomycetes. Finally, the fact that arid soil communities exhibit lower H’ values than non-arid soil communities reflects the relevance of the particular physicochemical/environmental features of arid soils to drive the bacterial community structure.

### Academic landscape and collaboration trends in arid soil research

To address whether there is an association between the sampled sites and the country affiliation of the researchers, we plotted in the global map, represented by country colour, the number of main affiliations by country of each researcher included in this systematic review (Fig. 2). The results indicate that the country with most participation was the United States, followed by China, Chile, Australia, and Germany. Nearly all these countries contain arid environments within their continental territory; therefore, we could explain the higher participation as an interest in the close-term effects of desertification. However, Germany and some other countries without arid environments are participating in research focused on the microbiology of arid soils using NGS strategies. In contrast, many countries with large dry surfaces, as the Sahara and Namibia Deserts have no participation.

**Fig. 5:**
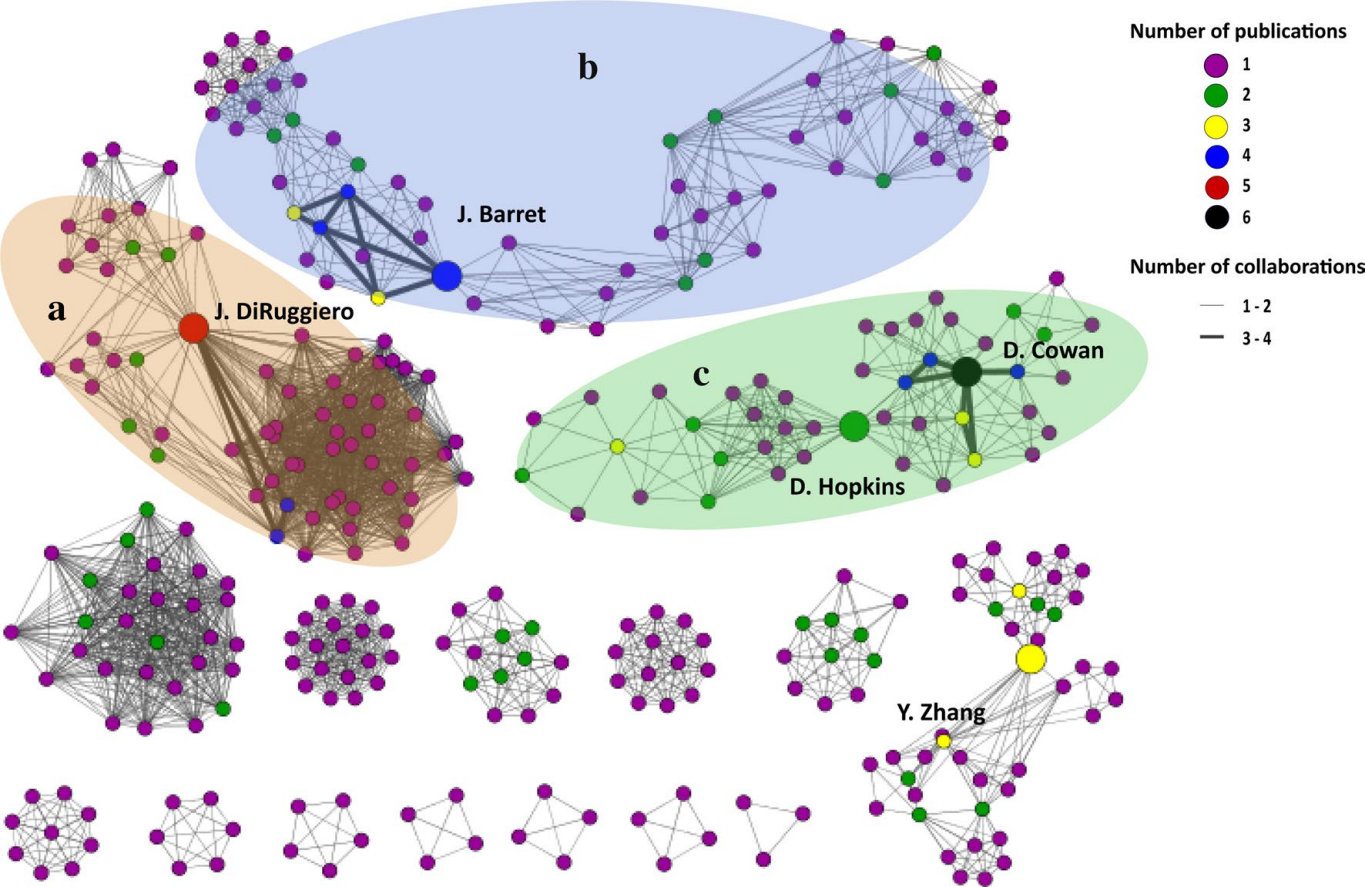
Arid soil microbiology co-authorship network. Each node represents an author publishing in the articles included in this review. The network was created using Cytoscape software [36] by entering the title of the article in the PubMed query bar within the Social Network App [22]. Node size represents the cumulative number of the author’s publication citation counts as automatically retrieved from PubMed based on the set of publications associated with the node (the count only includes citations of publications that are in PubMed Central). Thickness of the edges connecting the nodes represents the number of publications the two authors have published together. Double size nodes indicate the author has more than 50% of the betweenness centrality values in the network, also called keystone authors. **a**–**c** represent the three largest module-networks

To identify research groups focused on the study of microbial communities of arid soils and the relations between authors, we constructed a co-authorship network using Cytoscape v3.7.1 Software (Fig. 5). Using the NetworkAnalyzer tool, we were able to extract data to describe the network. A total of 333 authors and 2663 collaborations were identified and used to create the network, which resulted in 16 module-networks. The network density, defined as the portion of the potential connections in a network that are actual connections, was 4.8% indicating there are fewer collaborations than possible. The number of collaborations of each author did not exceed six events with other authors. When we calculated the Neighbour connectivity distribution, we observed that most authors have collaborations with one or two authors, while a few authors have various collaborations (high neighbour connectivity). From the 333 authors, we calculated the betweenness centrality value, defined as the influence a node/author has in the flow of information in a network, and five were classified as keystone author for having the 50% higher values of betweenness centrality.

The analysis of the sub-networks shows that the biggest module-network (A) was composed of 66 authors, while the second (B) and third (C) largest modules were composed of 61 and 45 authors, respectively. Interestingly, researchers associated with module-networks A and B have developed their research primarily in the Chilean Atacama Desert and the Chilean Antarctic territory. However, the B module network is led by North American and European researchers, who have not actively collaborated with Chilean researchers. Therefore, the larger research groups do not necessarily carry out their research in their country of origin and do not necessarily maintain active collaborations with researchers affiliated to the country in which the research is carried out. These results altogether suggest that this field of study can be exploited through a greater number of national and/or international collaborations.

## Discussion

The objectives of this review were (1) to identify and describe the state-of-the-art of bacterial communities in arid soils at a global scale, and address the effect that some environmental features may have on them; (2) to evaluate the editorial and co-authorship information of the researchers in this field. To address these aims, we performed a systematic review based on the PRISMA guideline, a methodology commonly used in biomedical and agronomical research areas [6, 7, 32, 38]. This approach gave us insight into the landscape of arid soil microbiology and permitted us to identify gaps in the body of knowledge through the analysis of the complete collection of articles focused on this topic.

To evaluate our search strategy, we calculated metrics such as sensitivity (75%), precision (4.5%) and NNR (22.2), which indicated that our search was good enough to find relevant articles but with low precision, according to Dieste and colleagues [13]. This means that our search strategy was lax enough to gather all, or most, relevant studies at the cost of reviewing 22.2 articles for every included one. These metric values are expected when the objective is to amass the complete set of studies of a defined research area.

The strategy of systematically searching for studies allows the standardized compilation and visualization of information that is not easily accessible otherwise. This approach allowed us to identify 66 articles that include samples from every arid dryland recognized on earth, with a good representation of both hemispheres. However, our results showed that, despite this distribution, there are sites over-represented (i.e. Antarctic Dry Valley, North China Deserts and the Chilean Atacama Desert). A plausible explanation can be the extreme environmental conditions displayed in these environments, and the biological implications that living under these conditions have. To survive, microorganisms have developed unique metabolic capacities, some of which are of interest to researchers in different areas [44]. This observation pinpoints the need to intensify the research in underrepresented arid environments, to have a thoroughgoing knowledge of the physicochemical and microbial characteristics of arid soils globally. Another observation that arises from this study is that over 60% of the arid soils sampled worldwide are cold and cool environments. With climate change, it is crucial to understand how microbial processes develop and change over time in these particularly vulnerable areas, and how this change will affect biogeochemical cycles [46].

The included articles in this review embody a database which could be robustly analysed in search of ecological patterns in arid soil communities. Though, this would require additional geographical, environmental, and physicochemical data (ideally raw data), which is absent from many studies. This issue is critical in extremely arid soils, which display a wide diversity of microenvironments [1] and in which physicochemical features are strongly related to bacterial community structure [16]. This limitation shows the need for future studies to make available as much raw data as possible per sample, as occurs in other fields of research.

To address this need and reduce the gap of knowledge, we suggest the following report guideline: (1) geographical data of sampling sites (i.e. coordinates and altitude), (2) environmental and physicochemical data (i.e. biome type, soil type, soil texture, sampling depth, soil salinity, mean annual temperature, mean annual precipitation, aridity index, soil pH, soil conductivity, soil evapotranspiration, total organic carbon percentage, and total nitrogen percentage; [3, 30, 40], (3) methodological data (i.e. DNA extraction protocols, sequencing platform, 16S rDNA gene region sequenced, algorithms of read analysis, taxonomic database and versions used) and (4) sequencing results data (i.e. relative abundance of OTUs and raw data, for instance in SRA database_NCBI).

Although environmental, geographical and physicochemical data were not reported in every article, as a result of this study, it was possible to identify some interesting trends of bacterial communities in arid soils. Despite the fact that all sites are classified as “arid” according to MAP, there is a wide variability of geographic, environmental and physicochemical factors within and between soils. For instance, altitude, MAT, soil pH, and soil conductivity vary markedly within and between arid environments, while TOC and TN did not. This observation was expected since MAP significantly correlates with TOC and TN [5], and arid soils have limited vegetation and biological activity [39], resulting in low levels of organic carbon and nitrogen available [33, 45].

Bacterial diversity was analysed by contrasting the Shannon diversity index with environmental variables. We observed that pH and electric conductivity had no noticeable effect over bacterial diversity in arid soils, at least in cold environments as the Dry Antarctic Valleys. Environmental microbiology literature has considered pH as one of the main drivers of microbial soil diversity in different soil types [15]. However, the absence of a relationship between diversity and pH in arid soils has recently been reported by other authors [25, 30], who observed that relative humidity, electric conductivity and temperature of the soil, are stronger determinants for bacterial diversity in the Atacama Desert. Our results allow us to extend this observation to other hot and cold arid environments and confirm the relevance of both temperature and water availability over the microbial structure of arid soils.

Regarding the microbial abundance evaluated by NGS technology, our results indicate that arid environments are dominated by phyla Actinobacteria, Proteobacteria, Bacteroidetes, Acidobacteria and Firmicutes, while other such as Marinimicrobia, Chlamydiae, Tenericutes and Saccharibacteria are absent. By means of other non-NGS technologies, some authors have also described Actinobacteria as the dominant phylum in arid soils and its positive relationship with a decrease in relative soil humidity [9, 29]. In contrast, our results indicate a reduction in the abundance of Proteobacteria, Cyanobacteria and Planctomycetes when decreasing the soil humidity. Contrasting microbial abundance against an increment of MAT, we observed an increase of Proteobacteria and Firmicutes; and a decrease of Acidobacteria and Bacteroidetes (Fig. 4). This last phylum has been studied by Yao et al. [47] who reported a high abundance of Bacteroidetes in the Inner Mongolian cold steppe. Similarly, Kumar et al. [23] showed that Bacteroidetes were dominant in cold environments but not in the cooler sites studied, suggesting that, although Bacteroidetes thrive in cold environments, the temperature is not the only feature that makes them prosper in cold arid soils. Arid environments included in this review harboured less abundancy of Cyanobacteria than other soil microbiomes. This could be explained since we selected bulk soil samples and opted out Biological Soil Crust (BSC) samples, in which soil particles are aggregated by the cohesive quality of some Cyanobacteria (Jayne Belnap [18, 20].

Finally, the academic collaborations landscape analysis, indicate that the social networks have both a low number of authors and degree of collaboration according to the criteria of Fagan et al. [14] and Campbell et al. [8]. Furthermore, the network density was very sparse, which indicates low connectivity. Despite, each of the two main module-networks (A and B) contain one keystone author (J. DiRuggiero and J. Barred, respectively), while the module-network C contains two keystone authors (D. Cowan and D. Hopkins). Therefore, these authors have formed research groups in this study area and have impacted the development of current knowledge of bacterial communities associated with arid soils. Undoubtedly, this knowledge has been complemented by the significant contribution of other groups that form individual networks around the world. A greater number of national and/or international collaborations would link these individual groups to the large network of interactions, strengthening collaborations worldwide.

## Conclusions

The present systematic review allowed us to compile and visualize all or most of the current information related to the bacterial communities of arid soils through an integrative approach. This strategy permitted us to identify articles that include samples from every arid dryland recognized on Earth, noting that there are over and underrepresented sites among the studies.

Our results reaffirm some observations reported by other authors, like bacterial communities from arid soils being different from those of non-arid soils. In particular, we can indicate that arid soils display a higher abundance of Actinobacteria and lower abundance of Proteobacteria, Cyanobacteria, and Planctomycetes, comparatively. This microbial structure seems to be strongly modulated by MAP and MAT and not by pH in arid soils.

We also observed that despite that all sites studied were classified as “arid” according to MAP; there is a wide variability of geographic, environmental and physicochemical features within and between soil sampling sites, highlighting that many factors could explain the differences observed in the composition of bacterial communities among arid soils. However, to increase accuracy when searching for relationships between the microbiome and abiotic factors, it is necessary to count on the information proposed in our report guideline, which is useful for further analysis (e.g. meta-analysis at global-scale).

Finally, the analysis of the author’s network showed that, currently, there is a low degree of connectivity and collaboration in this research topic. In the meantime, the phenomenon of desertification is increasing due to global climatic change, hence it is crucial to understand how microbial processes develop and change in arid soils, and how these changes affect biogeochemical cycles. Considering this is a global challenge, our analysis emphasizes the need to increase the connectivity and collaboration between the research groups worldwide.

## Supplementary information

**Additional file 1.** Editorial, environmental, geographical and microbial community extracted data.

**Additional file 2.** Summary of the metadata extracted in Additional file 1.

**Additional file 3.** Reported environmental information, arid soil bacterial community structure and plotted editorial data.

## Data Availability

All data generated or analysed during this study are included in this published article [and its Additional files].

## Abbreviations

PRISMA: Preferred Reporting Items for Systematic Reviews and Meta-Analyses
OTUs: Operational Taxonomic Units
UNCCD: United Nations Committee to Combat Desertification
MAP: Mean Annual Precipitation
PET: Potential Evapotranspiration
WOS: Web of Science
I.M.: Isidora Morel
F.M.: Felipe Maza
J. V-D.: Javiera Vásquez-Dean
R.P.: Rodrigo Pulgar
M.G.: Mauricio González
GPS: Global Positioning System
MAT: Mean Annual Temperature
TN: Total Nitrogen
TOC: Total Organic Carbon
EC: Conductivity
H’: Shannon Diversity Index
BSC: Biological Soil Crust
HA: Hot arid
CA: Cold arid
