# Supplementary material for: Microbial communities from arid environments on a global scale. A systematic review
Source: Biol Res. 2020 Jul 6;53:29. doi: 10.1186/s40659-020-00296-1 (PMC7336661; doi:10.1186/s40659-020-00296-1)
Supplement: Supplementary file 3 — Additional file 3. Reported environmental information, arid soil bacterial community structure and plotted editorial data. [file 40659_2020_296_MOESM3_ESM.docx]

**Additional file 3**


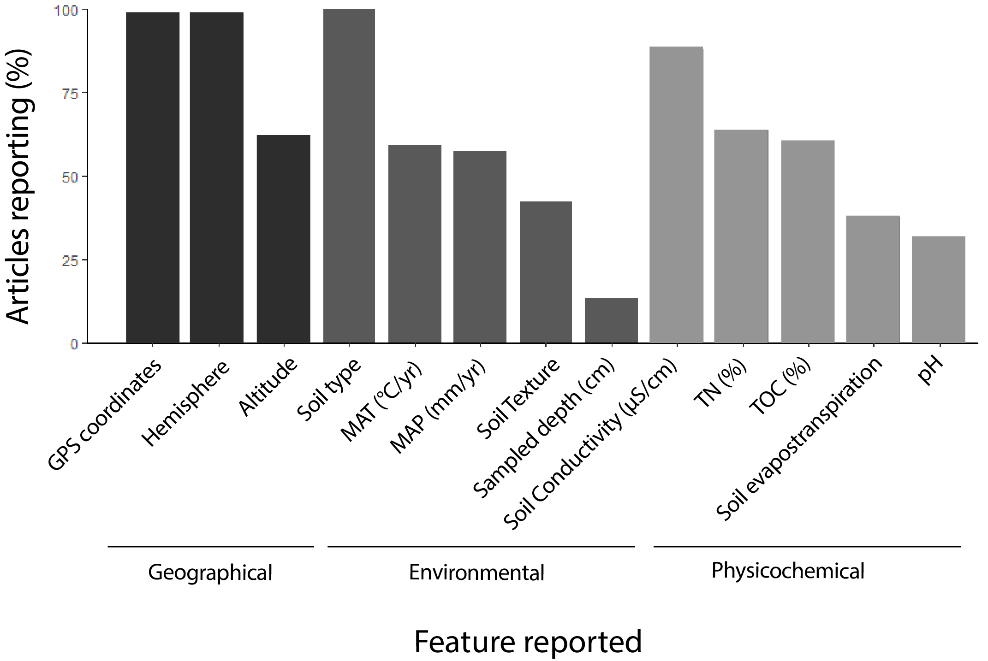


**Additional file 3: Figure 1: Reported environmental features:** Percentage of articles included (n=66) in this systematic review that explicitly report each environmental feature from their sampling sites. Data was extracted from each article, missing data was registered as #N/D, percentage of reported data was used to plot using ggplot package in R software.


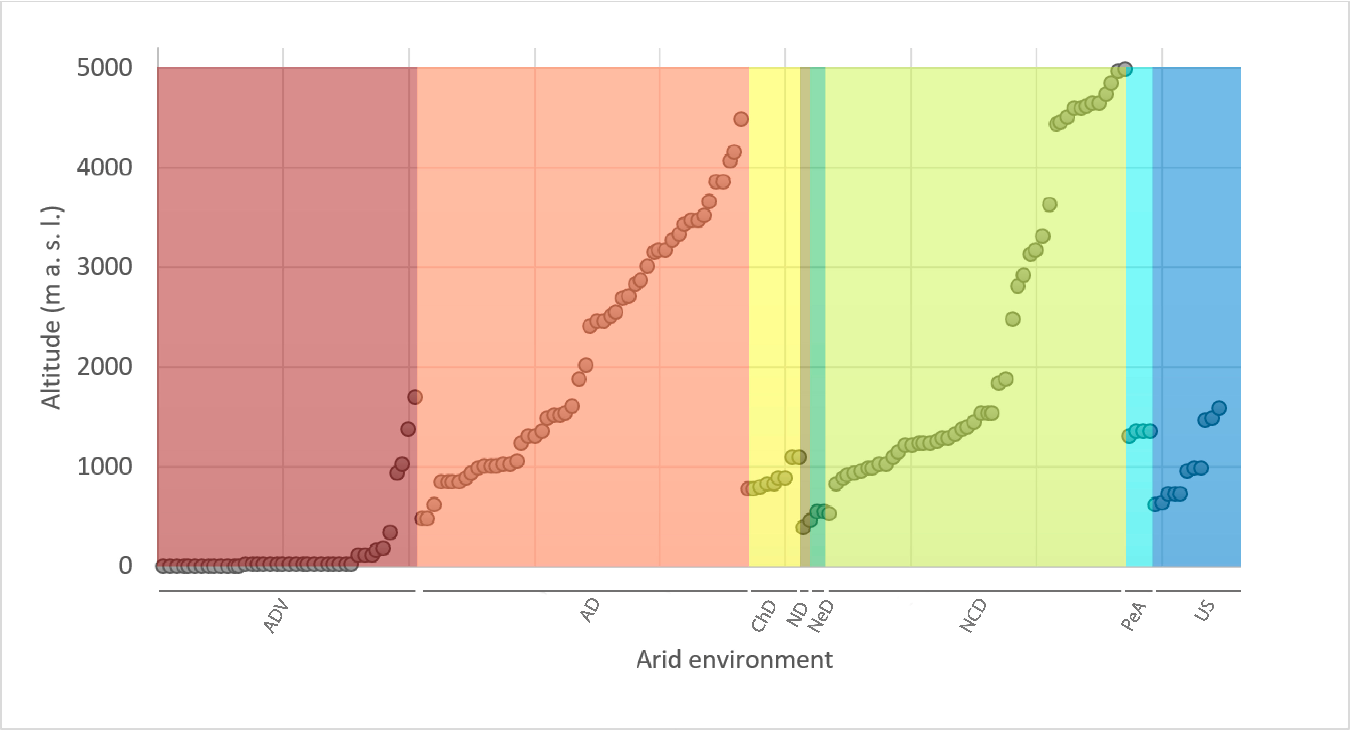


**Additional file 3: Figure 2a:** **Altitude profile of sampled environments:** Each dot represents a sampled site from each environment included in this systematic review (n=169). Samples are arranged following the increase of altitude (m a.s.l.) values in each location. Abbreviations: WA, Western Antarctica; AP, Argentinian Patagonia; AD, Atacama Desert; ChiD, Chihuahuan Desert; US, South Western United States; ND, Namibia Desert; PeA, Princess Elisabeth Station Antarctica; ChD, Chad Desert; MD, Moroccan Desert; AlgD, Algerian Desert; TD, Tunisian Desert; NED, North Egyptian Desert; NeD, Negev Desert; Jdn, Jordan arid soils; QD, Qatari Desert; IrD, Iran Desert; ID, Indian Desert; KshD, Kazakhstan Desert; NCD, North China Deserts; SAD, South Australia Desert; ADV, Antarctic Dry Valleys; Nrw, Norway.


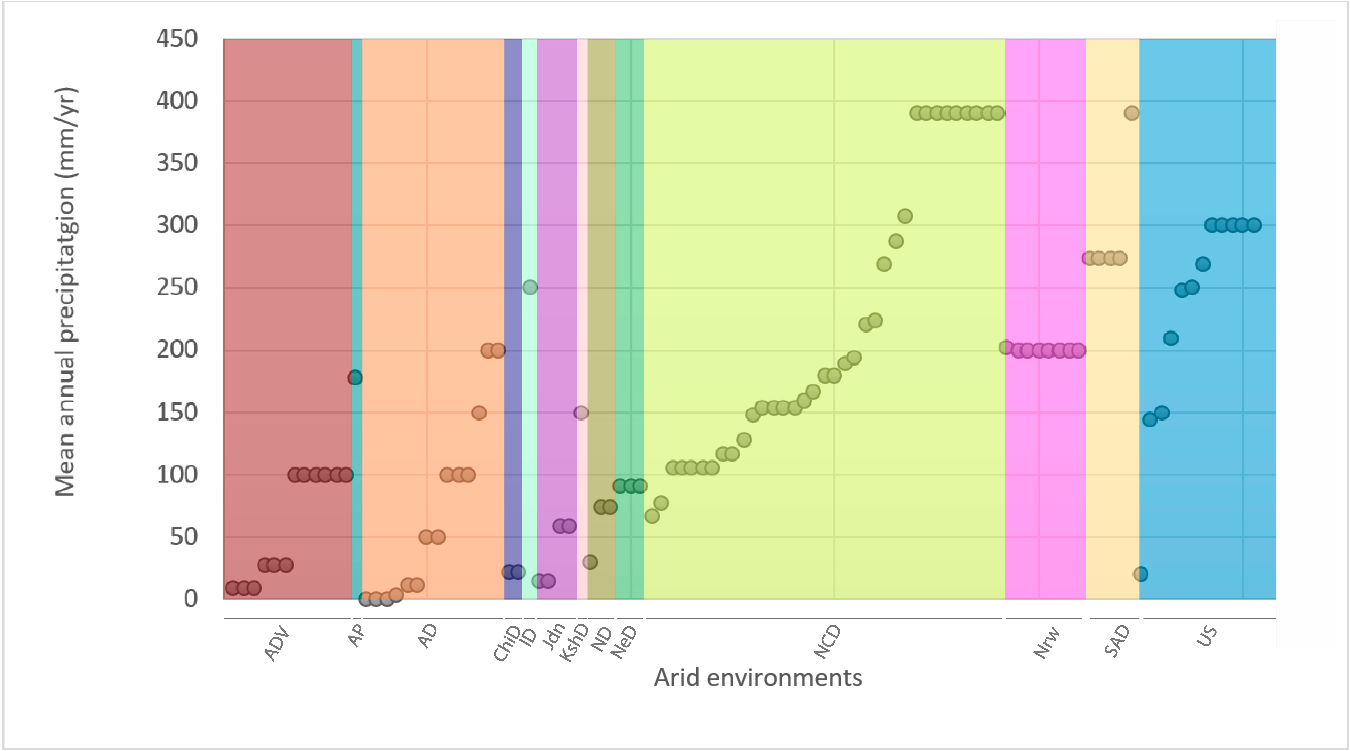


**Additional file 3: Figure 2b: MAP profile of sampled environments:** Each dot represents a sampled site from each environment included in this systematic review (n= 102). Samples are arranged following the increase of MAP values in each location.


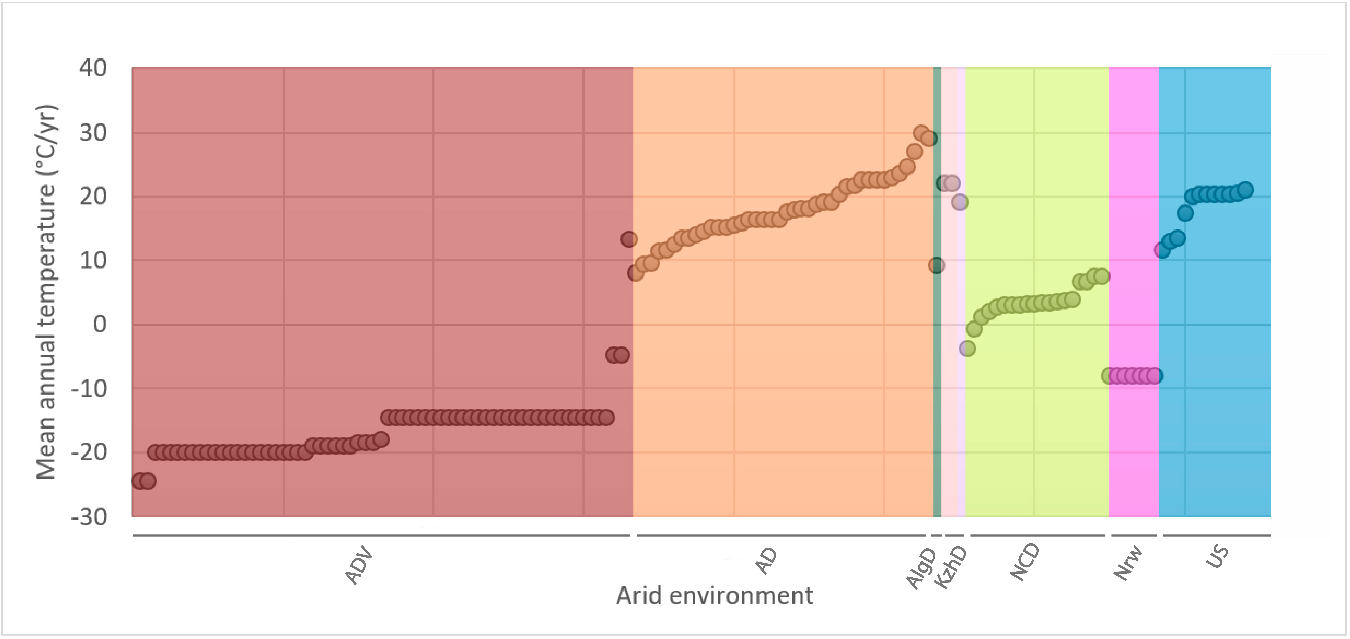


**Additional file 3: Figure 2c: MAT profile of sampled environments:** Each dot represents a sampled site from each environment included in this systematic review (n=148). Samples are arranged following the increase of MAT values in each location.


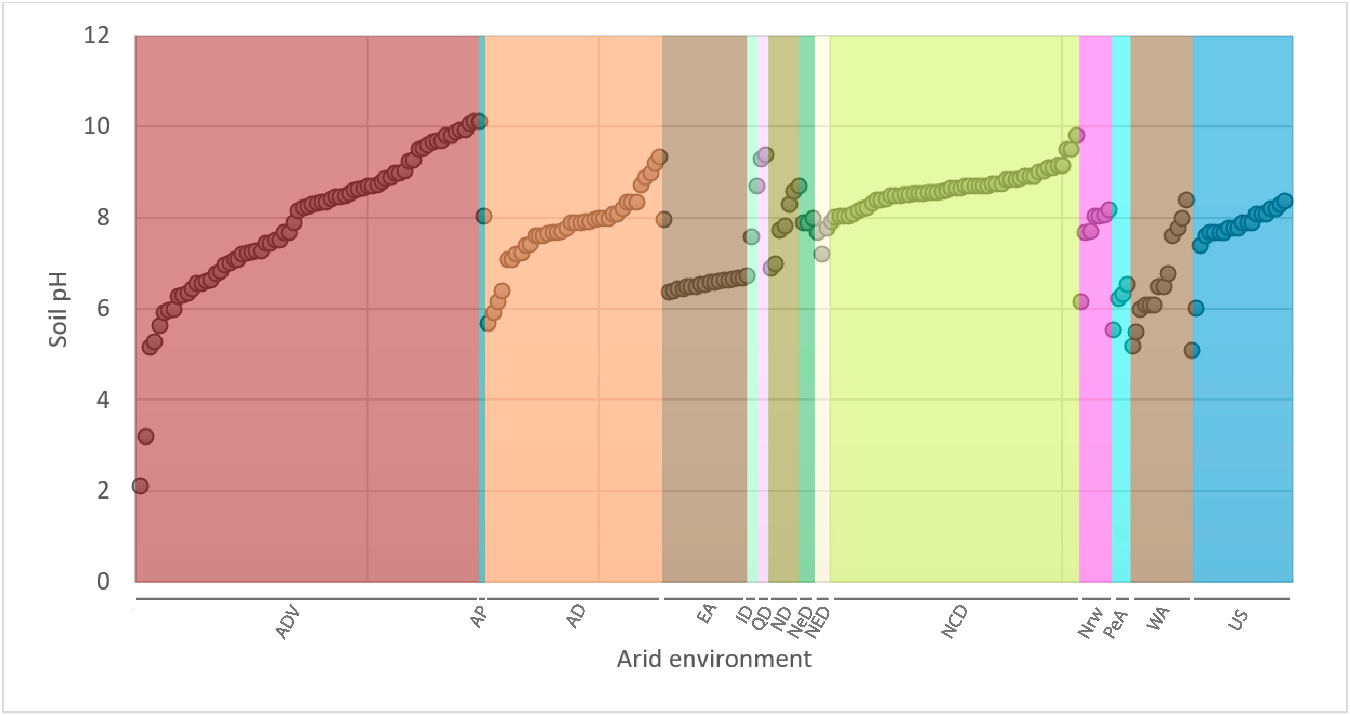


**Additional file 3: Figure 2d: pH profile of sampled environments:** Each dot represents a sampled site from each environment included in this systematic review (n=248). Samples are arranged following the increase of pH values in each location.


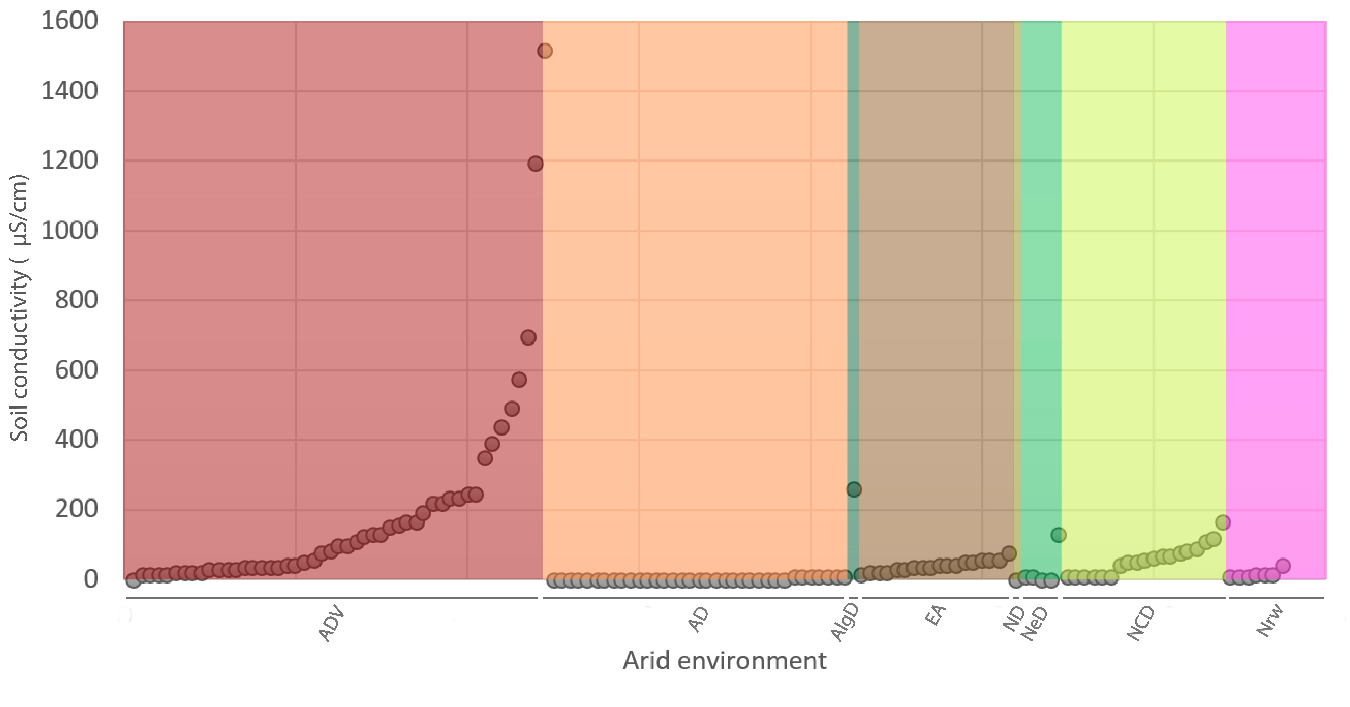


**Additional file 3: Figure 2e: Soil conductivity profile of sampled environments:** Each dot represents a sampled site from each environment included in this systematic review (n=135). Samples are arranged following the increase of soil conductivity values in each location.


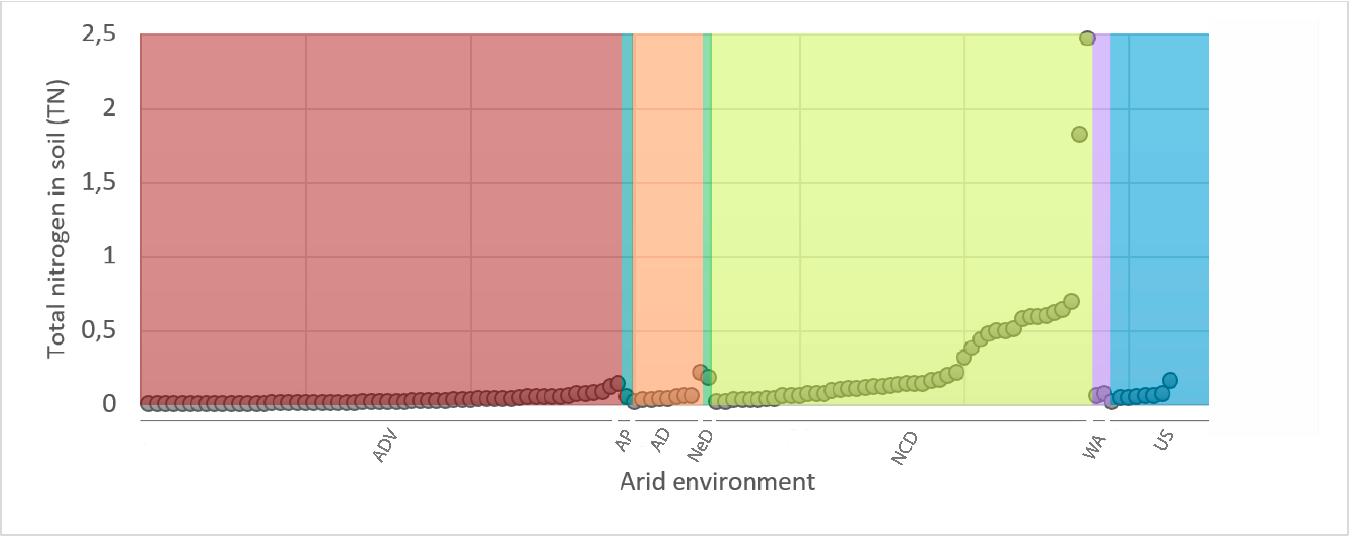


**Additional file 3: Figure 2f: Total nitrogen in soil profile of sampled environments:** Each dot represents a sampled site from each environment included in this systematic review (n=125). Samples are arranged following the increase of total nitrogen (TN) values in each location.


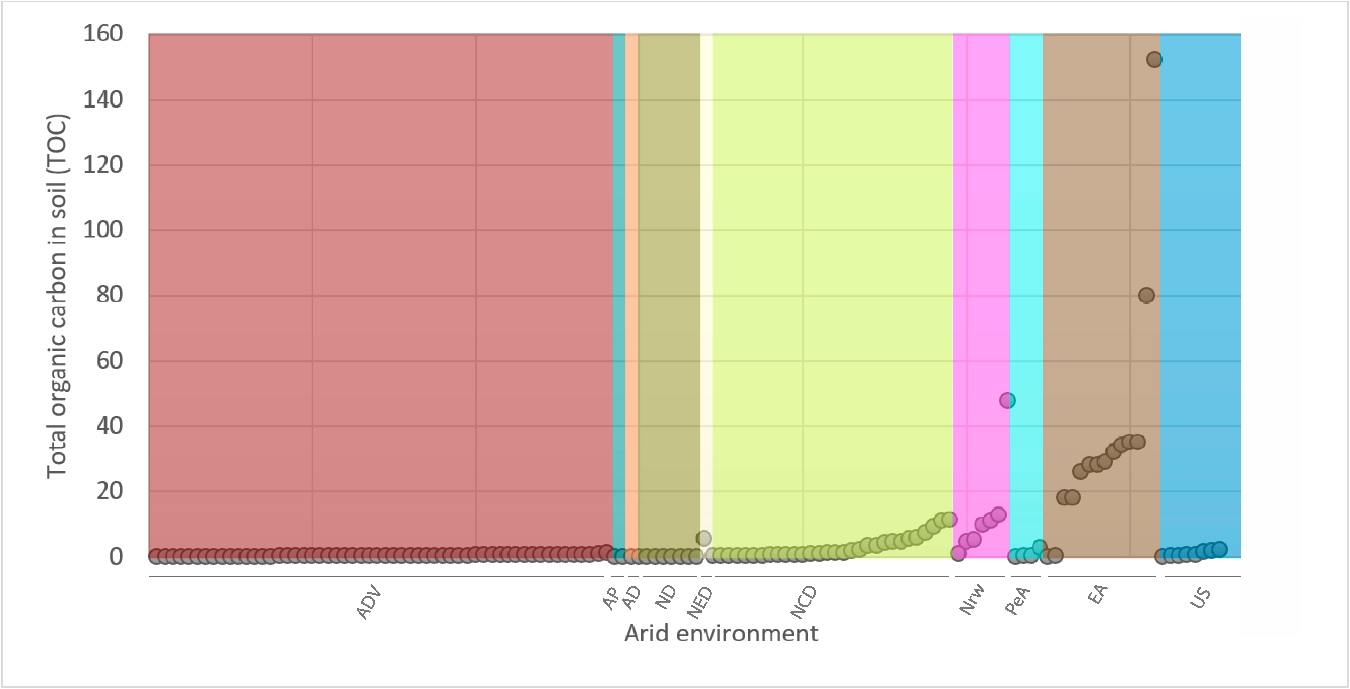


**Additional file 3: Figure 2g: Total organic carbon in soil profile of sampled environments:** Each dot represents a sampled site from each environment included in this systematic review (n=131). Samples are arranged following the increase of total organic carbon (TOC) values in each location.


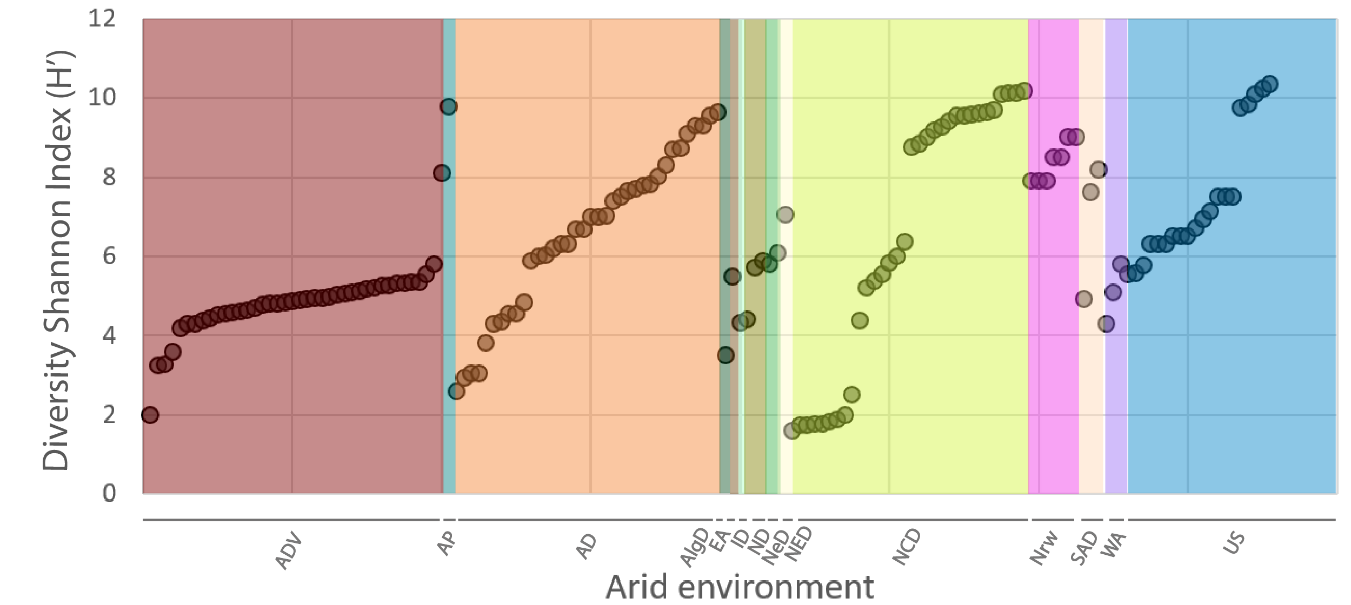


**Additional file 3: Figure 2h: Diversity Shannon Index (H’) from soil bacterial communities of sampled environments:** Each dot represents a sampled site from each environment included in this systematic review (n=151). Samples are arranged following the increase of Diversity Shannon index (H’) values in each location.


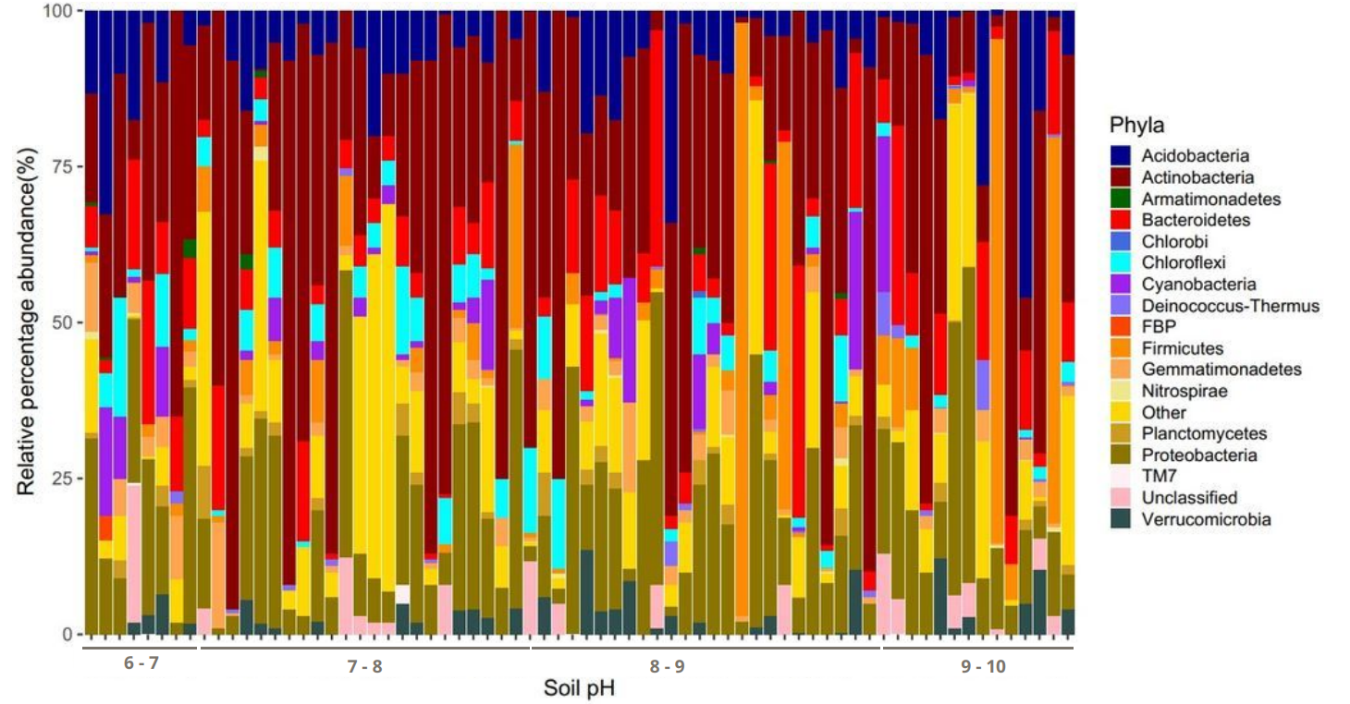


**Additional file 3: . Figure 3: Community structure of arid soil samples in relation to pH.** Vertical bars represent a sample from the articles included in this systematic review. Bars indicate relative abundance colored by bacterial phyla, sorted in ascending order of pH value (from 6.1 to 9.95) reported in each corresponding article. Plots where generated using ggplot2 package in R software.


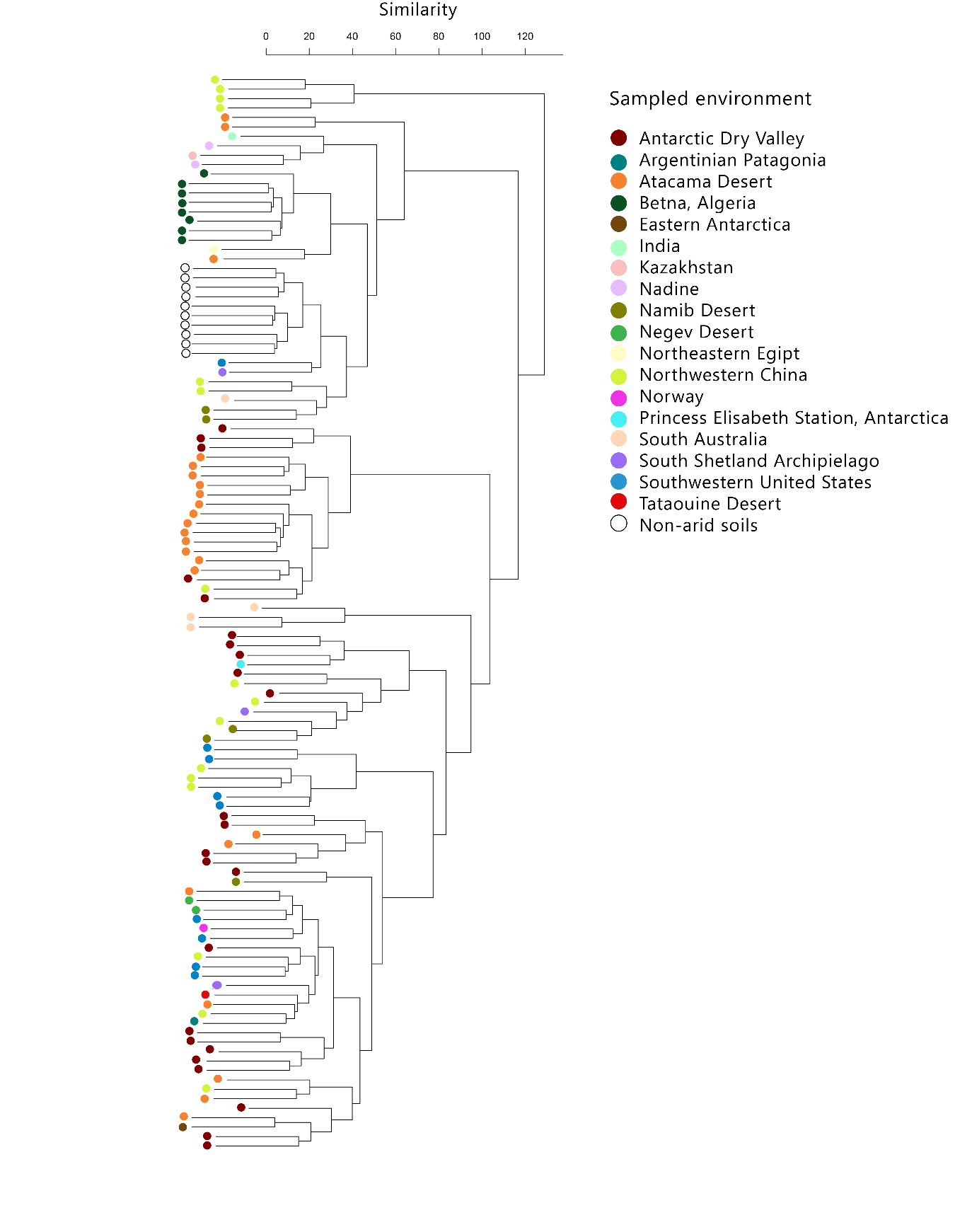


**Additional file 3: . Figure 4: Hierarchical clustering of sampled sites by environment according to community relative phyla abundance.**


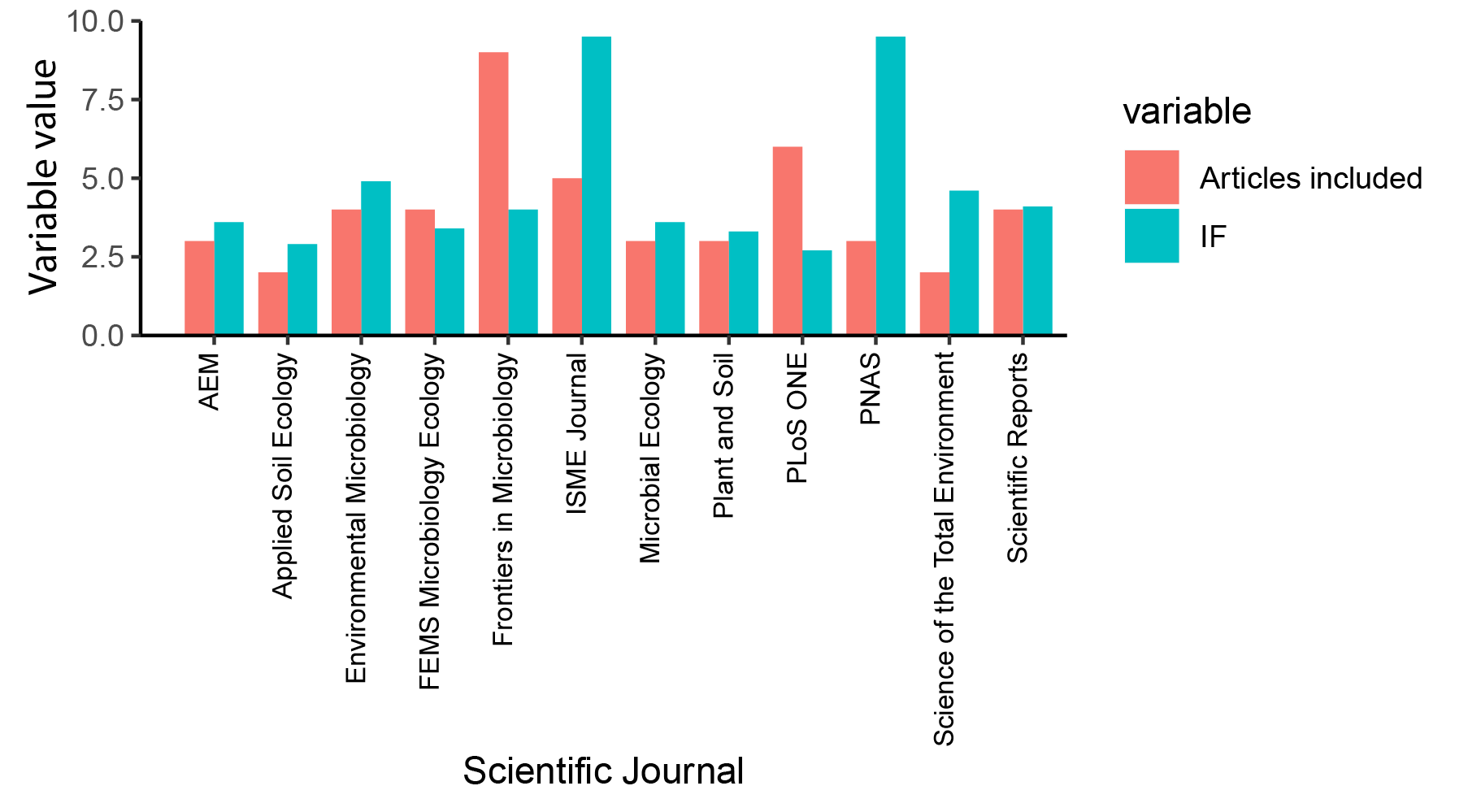


**Additional file 3: . Figure 5: Journal publishing and year of articles included in this review.** Double factor bar plot with publications by journal included in this systematic review with the correspondent impact factor (IF) 2018 of each journal.
